# The evolution of spatial ordering of oil drops fast spreading on a water surface

**DOI:** 10.1038/ncomms8189

**Published:** 2015-05-22

**Authors:** Daigo Yamamoto, Chika Nakajima, Akihisa Shioi, Marie Pierre Krafft, Kenichi Yoshikawa

**Affiliations:** 1 Department of Chemical Engineering and Materials Science, Doshisha University, Kyoto 610-0321, Japan; 2 Fluorinated Soft Matter Group, Institut Charles Sadron (CNRS), University of Strasbourg, 23 rue du Loess, Strasbourg 67034, France; 3 Laboratory of Life Physics, Faculty of Life and Medical Sciences, Doshisha University, Kyoto 610-0394, Japan

## Abstract

The design of dynamically self-assembled systems is of high interest in science and technology. Here, we report a unique cascade in the self-ordering of droplets accompanied by a dewetting transition. The dynamic self-emergent droplets are observed when a thin liquid layer of an immiscible fluorocarbon oil (perfluorooctyl bromide, PFOB) is placed on a water surface. Due to the gradual evaporation of PFOB, a circular PFOB-free domain appears as a result of a local dewetting transition. A circular pearling structure is generated at the rim with the growth of the dewetting hole. As the next stage, linear arrays of droplets are generated in a radial manner from the centre of the hole. These one-dimensional arrangements then evolve into two-dimensional hexagonal arrays of microdroplets through collective rhythmical shrinking/expanding motions. The emergence of such dynamic patterns is discussed in terms of the nonlinear kinetics of the dewetting transition under thermodynamically dissipative conditions.

## Abstract (web summary)

The spreading of liquids on water can lead to complex drop assemblies, but none of them so far exhibits coordinated dynamics. Here, Yamamoto *et al*. observe a dance of insoluble oil drops on a water surface evolving from linear to hexagonal arrays, due to dewetting transition and evaporation of oil.

## Graphical abstract

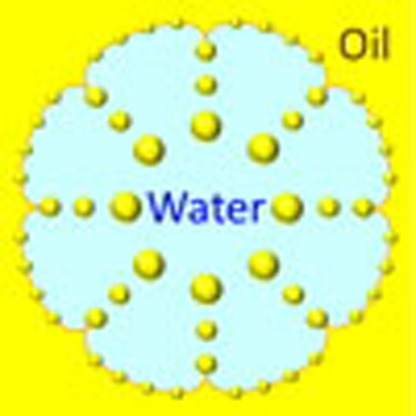

The dynamic self-assembly of locally interacting individual entities to form patterns with higher-level structures and complexity plays a fundamental role in nature, science and technology^1^,^2^,^3^. In both living and non-living systems, spatiotemporal patterns are frequently mediated through various types of interfaces under thermodynamically open conditions. Collective motion, which is ubiquitous in ensembles of both primitive organisms and evolved animals, offers fascinating examples of spatiotemporal patterns, such as those formed by, for example, colonies of bacteria^4^, schools of fish^5^, flocks of birds^6^, ungulate herds and even human crowds^7^, in which individuals can behave in synchrony. Simple abiotic synthetic systems that are capable of autonomous coherent motion are useful for unveiling the underlying mechanisms of biological collective motion. Since the earliest reports of the ‘camphor dance'^8^,^9^, the autonomous motion of solid or solid-like particles has attracted interest in associated phenomena, including micromotors such as catalytic particles^10^,^11^, AgCl particles^12^, self-oscillating responsive polymer gels^13^, nematic liquid crystal phases^14^ and magnetic disks^15^. With regard to liquid systems, droplets of partially water-soluble alcohols^16^, aniline^17^ and dichloromethane^18^, when dissolved at the surface of water, have been shown to display complex shapes and self-motility. None of these systems, however, exhibit coordinated droplet dynamics, which could lead to synchronous collective movement of the ensemble. Fluorocarbons offer a combination of unique properties, including quasi-insolubility in water, rapid spreading on water surfaces, low surface tension and high volatility with respect to molecular weight, which makes them effective building blocks for the design of self-assembling systems.

We describe here a series of hitherto unreported phenomena that occur over time during the dewetting of thin films of a liquid fluorocarbon (perfluorooctyl bromide (PFOB), C_8_F_17_Br) spread on water. These phenomena include the spontaneous formation of similarly sized and regularly spaced humps at the fluorocarbon's dewetting front, the chain ejection of linear arrangements of microdroplets from these humps and the transition from these one-dimensional (1D) arrangements to two-dimensional (2D) hexagonal arrays through a shrinking/expanding rhythmical behaviour. These events are made possible by the distinctive properties of the selected fluorocarbon, which is essentially insoluble in water, yet highly volatile and fast spreading. The observed unique time-dependant dynamic coherent translational motion that leads to regular ordering of the droplets is interpreted solely on the basis of a dissipative effect due to evaporation of the fluorocarbon.

## Results

### A series of dynamical events of a fluorocarbon droplet

**Figure 1:**
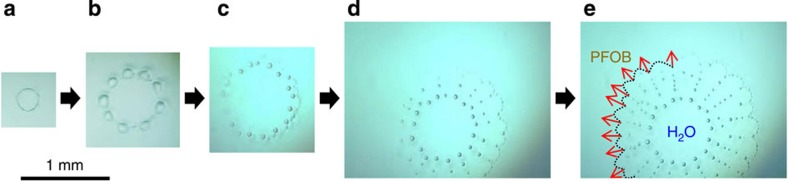
Generation of droplets pearling accompanied by a dewetting transition. (**a**) A thin film of PFOB floating on a water surface yields water holes decorated with pearling humps caused by the dewetting transition of the film, accompanied by the slow evaporation of PFOB. (**b**) A critical point is reached when, during the growth of the hole, the PFOB rim acquires a smaller curvature than that of the humps. (**c**) This triggers a rhythmical chain ejection of microdroplets from the humps. (**d**,**e**) After the first ejection, humps that are smaller than the initial humps are created and ejected repetitively. The black dotted line and red arrows represent the contact line between PFOB and water, and the direction in which the contact line recedes, which also indicates the direction of the growing hole of water. Note that the images (**a**,**b**) and (**c**–**e**) were obtained from different holes, due to technical reasons.

**Figure 2:**
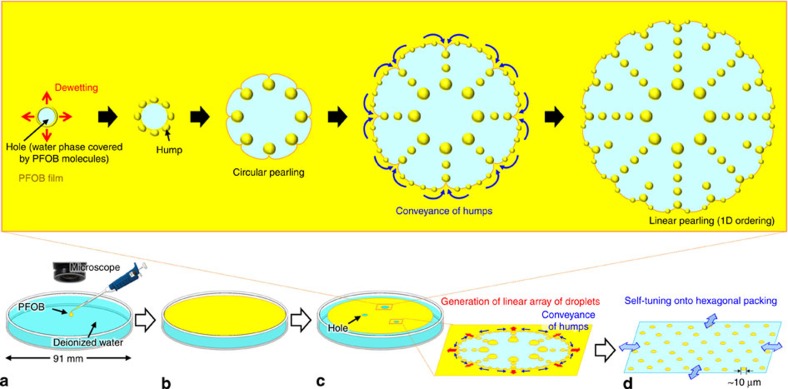
Spontaneous dynamic behaviour of PFOB spread on water. (**a**) PFOB is pipetted onto a water surface. (**b**) Immediately after contact with water, it spreads to form a thin film. On the basis of the injection volume (8 μl) and the surface area of water (∼65 cm^2^), the thickness of the film can be estimated to be 1 μm. (**c**,**d**) As the PFOB gradually evaporates, the system exhibits characteristic pearling phenomena consisting of four successive non-equilibrium events; event I: a water hole is generated through the evaporation of PFOB molecules and the dewetting transition; event II: fluorocarbon humps are formed on the rim of the water holes, which causes circular pearling of the droplet array; event III: appearance of a linear pearling (1D array), where droplets are generated periodically; event IV: (**d**) a regular hexagonal 2D structure is formed over the entire water surface by rhythmical shrinking and expanding motion, and remains for a relatively long time as a stable pattern. Finally, the arranged droplets disappear within 20 s due to evaporation.

When a droplet of the selected liquid but volatile fluorocarbon, PFOB, is deposited on the surface of deionized water, it spreads immediately over the entire surface due to its positive spreading coefficient (+2.7 mN m^−1^)^19^. Surprisingly, the thin layer (film) of PFOB then spontaneously undergoes a series of dynamical events that occur in a cascade that culminates with the collective translation of ensembles of microdroplets (Figs 1 and 2; Supplementary Movie 1). Chronologically, these events involve (I) the formation of circular PFOB-free domains (water holes) in the PFOB film caused by its dewetting; (II) the progressive upheaval of the rim of these holes (which is the three-phase circular contact line) to form regularly distributed humps of PFOB; (III) the rhythmical cascade ejection of microdroplets from these humps, creating radial 1D alignments; (IV) the collective translational motion of the microdroplet ensemble resulting in the formation of regular 2D hexagonal patterns.

To the best of our knowledge, this is the first report of spontaneous transformation from a 1D array to a 2D array of microdroplets through coherent motion. This dynamic behaviour strongly differs from previously reported static orderings, such as the formation of hexagonal patterns of crystallized polymer aggregates, generated by a stick–slip sequence of the contact line during dewetting of the polymer solution on a solid substrate^20^, or the hexagonal lattices of monodisperse crystalline nanodomains formed by (perfluoroalkyl)alkanes on water and solid substrates^21^,^22^,^23^.

### Formation of liquid fluorocarbon humps around water holes

**Figure 3:**
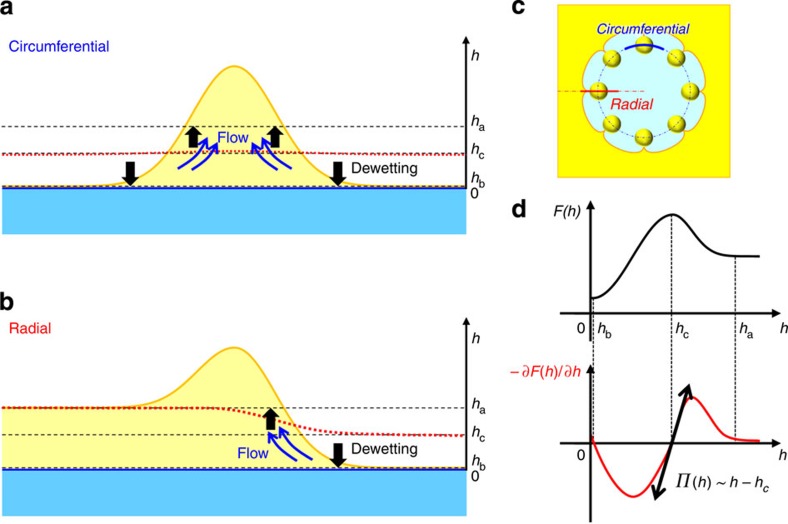
Scheme of the dewetting transition leading to pearling instability. The dewetting transition first causes circular pearling and then linear pearling. (**a**,**b**) Schematic representations of the side view along the circular and radial lines in a hole, which correspond to the blue and red lines in the top view (**c**), respectively. *F*(*h*) is the free energy of a PFOB film per unit surface area as a function of film thickness *h*, and the film thickness exhibits bistability with a maximum at *h*=*h*_c_ (**d**)^24^. If we begin the experiment with the entire surface covered by a sufficiently thick PFOB film, *h*>*h*_a_, thinning of the film due to gradual evaporation causes instability and, accompanied by the dewetting transition, a hole appears and grows. Depending on the time development of the hole, the dewetting transition generates circular and linear pearling, as depicted in the figure. A more detailed explanation of the pearling instability in these experiments is given in Supplementary Note 1.

At the very initial stage of the experiment, the existence of a thin film of PFOB covering the entire surface of water demonstrates that the spreading coefficient of PFOB is indeed positive (Fig. 2b). During the thinning process caused by the evaporation of PFOB, the film of PFOB becomes unstable when it becomes thinner than a critical thickness, which induces a dewetting transition. A circular water hole is then spontaneously generated within the film (Figs 1 and 2c). Note that the surface of the hole is not pure water, but rather is covered by PFOB molecules (nanolayer) as described later. The formation of holes in a thin oil film, as well as fragmentation, fingering, pulsation and/or autonomous motions, is well known when organic solvents such as 1-butanol and 3-octanol^16^, aniline^17^ or dichloromethane^18^ are spread on water. These phenomena are interpreted in terms of dewetting instability^24^. The behaviour of our system is original. As the hole grows, the rim of the PFOB film starts upheaving, resulting in the formation of PFOB humps that are regularly distributed along the periphery of the hole. To the best of our knowledge, the generation of regularly spaced humps in receding organic solvent fronts has not been observed previously. Actually, we verified that a film of a volatile hydrocarbon solvent, cyclohexane, exhibits a monotonous evaporation process, with the formation of water holes that grow without the formation of ordered humps until the film disappears (Supplementary Movie 2). The formation of these humps at the rim is attributable to the bistability of the PFOB film height *h* on the water surface. It can be described as a pearling instability (Fig. 3). The occurrence of the dewetting transition implies that the local free energy *F*(*h*) of the film exhibits a maximum at the film's critical height *h*_c_. By considering the intrinsic property of the dewetting transition, one can expect that the disjoining pressure 
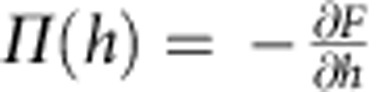
 is positive or negative when *h* is above or below *h*_c_, respectively. Thus, we can expect that the kinetics of the change in height can be given in a first-order approximation as 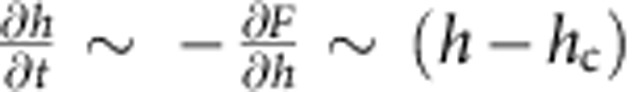. Here, the term 
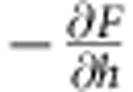
 corresponds to the disjoining pressure *Π*(*h*). Thus, near the critical height, the disjoining pressure exhibits symmetry with respect to *h* as follows: *Π*(*h*)∼(*h*−*h*_c_). Along the dewetting line, the thin film exhibits a severe instability attributable to the bimodality of the free energy depending on the depth of the film, that is, positive/negative disjoining pressure. Therefore, the disjoining pressure symmetry leads to the formation of humps (for a detailed theoretical interpretation, see Supplementary Note 1).

### 1D alignments through periodical ejection of microdroplets

The spatio-periodically arranged humps generated through pearling instability evolve to emit radial linear strings of microdroplets (Figs 1 and 2c). This behaviour is believed to occur as follows: as a consequence of hump formation, portions of the rim located in the vicinity of a hump acquire a highly negative curvature and tend to show an increase in height by attracting fluorocarbon oil molecules, thus further contributing to the instability of the film. This growing instability causes the repetitive, radial ejection of microdroplets from the humps, resulting in a time- and spatio-periodical 1D repeating pattern. Such 1D ejection of microdroplets to form a dendritic pattern is a unique phenomenon, and is different from all previous examples of the ejection of droplets, such as from drops of fatty alcohols deposited on water or of dichloromethane on solutions of cetyltrimethylammonium bromide^16^,^18^. In the latter case, the daughter droplets that are ejected radially from the mother drop disappear in less than 1 s due to rapid evaporation (vapour pressure: 47 kPa for CH_2_Cl_2_ versus 0.59 kPa for PFOB at 20 °C^19^) and the dissolution of CH_2_Cl_2_ in water. A seminal difference with our system is that the CH_2_Cl_2_ droplets radiate outwards from the periphery of the mother droplet onto a practically infinite water surface, while the fluorocarbon droplets propagate within a finite water hole that is confined by the fluorocarbon film. Therefore, our system uniquely provides self-confined conditions that allow the interactions between droplets to operate and generate close-packed patterns as described below.

**Figure 4:**
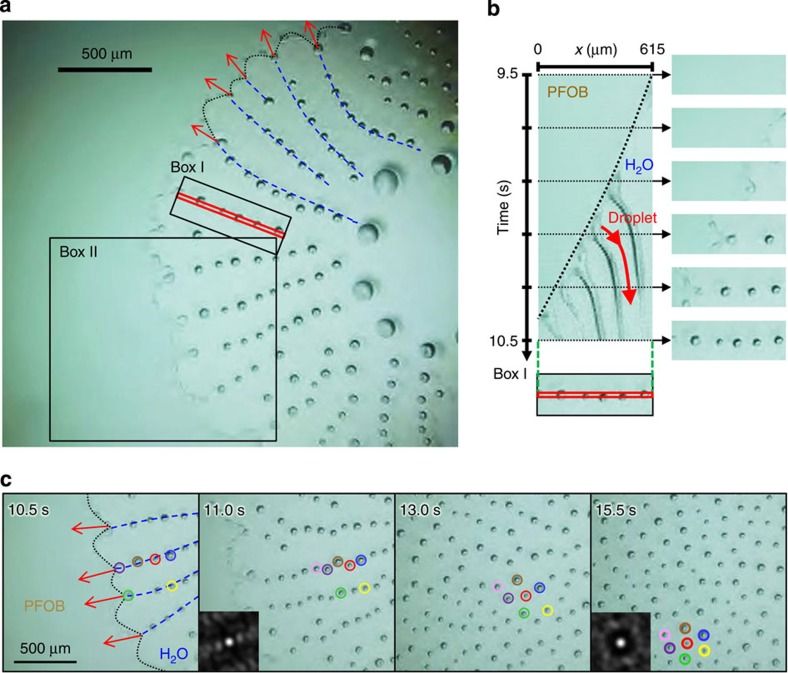
Transformation from 1D linear pearling to 2D hexagonal regularity. (**a**) Linear alignments of numerous regularly spaced droplets are produced by repetitive ejection. The contact line between PFOB and water (black dotted line) is a cycloid-like curve. Blue dashed lines represent typical trajectories of the projection points of the contact line. Red arrows show the direction in which the PFOB/water surface recedes. This demonstrates that microdroplets are ejected from projection points of the contact line to form 1D arrays. (**b**) Spatiotemporal diagram of 1D linear pearling is prepared along a red diagonal line in box I. (**c**) Spontaneous transformation from 1D linear pearling to a 2D hexagonal regular lattice in box II. To clarify the nature of the changes over time, the droplets are assigned different colours, indicating rearrangement from 1D to 2D hexagonal. Insets in snapshots at 11.0 and 15.5 s show the 2D Fourier transforms of the pattern. Times displayed on the figures represent the elapsed times after the injection of a PFOB droplet.

As the microdroplets are continuously ejected in the cascade, the humps surging at the rim and the ejected droplets become slightly smaller than those observed during the initial ejection period (Fig. 4). Figure 4b shows a spatiotemporal diagram of the repetitive microdroplet ejection pattern that propagates on a water hole. The time interval between two subsequent ejections is about 0.2 s, and the ejected droplets are aligned at almost identical spatial intervals. During repetitive ejection, the droplets move away from the contact line: the speed of their vectorial motion is maximal (∼0.5 mm s^−1^) just after ejection and decreases afterwards. This means that a repulsive force is at work between the droplets and the rim of the PFOB film. This repulsive force cannot arise from Marangoni flow^25^,^26^, because of the extremely low solubility of PFOB in water (∼5 × 10^−9^ mol l^−1^)^19^. Actually, the surface tension of water saturated with PFOB (measured after the deposition of a PFOB droplet at the bottom of a water-filled cell) is essentially identical (∼72 mN m^−1^) to the surface tension of pure water at 25 °C. Thus, we consider that the repulsive force is attributable to the outward 2D flow caused by dewetting transition together with gradual evaporation. The detailed mechanism is described in the next section.

### Collective motion of microdroplets generating 2D ordering

Our fluorocarbon oil/water system offers the remarkable capacity to spontaneously confine a population of droplets with a long lifetime in a closed space. As shown in Fig. 4c, the microdroplets that are initially aligned in a 1D pattern (10.5–13.0 s) rearrange to form a regular 2D hexagonal pattern (13.0–15.5 s). Insets in snapshots at 11.0 and 15.5 s show images of the 2D Fourier transforms of the pattern, demonstrating that the linear and hexagonal pattern is not local. The extent of the hexagonal ordering may appear modest on the figures because they are snapshots on a dynamic behaviour. Supplementary Movie 1 shows clearly that the droplets self-organize into a hexagonal ordering under large dynamical fluctuations, revealing that the self-organization is accompanied by rhythmical shrinking and expanding translational movements reminiscent of those of flocks of birds (Supplementary Fig. 1). Remarkably, contact between droplets and subsequent coalescence have never been observed to occur during their lifetime. This suggests that each droplet has an excluded area around itself that induces mutual repulsion. The existence of this excluded area is more easily appreciated at a lower temperature (10 °C). Each droplet shows rapid translational movement (∼8 mm s^−1^, corresponding to ∼200 body lengths per sec) (Supplementary Movie 3). When two droplets get close to each other (∼100 μm distance, only 3 body lengths), they start repelling each other. As a result, the direction of translational motion undergoes a rapid change, despite the droplet's high speed, to avoid collision.

**Figure 5:**
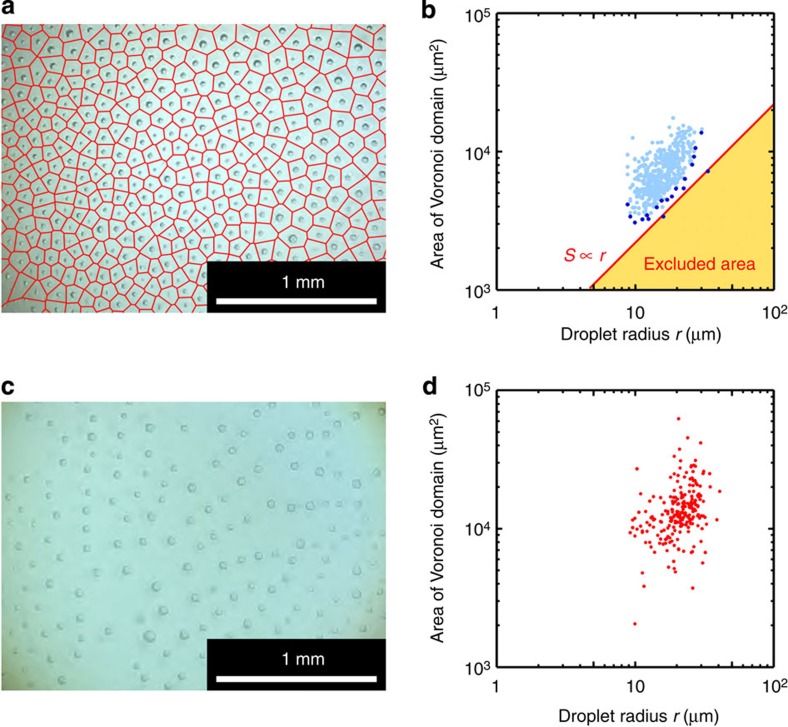
Rates of surface-spreading and evaporation of the fluorocarbon. (**a**) A Voronoi diagram of an example of a shrinking hexagonal pattern; seed points are set to be the centre of gravity of each droplet. (**b**) The relationship between droplet radius *r* and surface area of Voronoi domains *S*_Vor_, as obtained from **a**. Dark-blue circles show data points that have the minimum *S*_Vor_ in the particular range of droplet radius (interval of log-scaled radius: Δ(log_10_ *r*)=1/30). (**c**,**d**) Results of a control experiment where evaporation rate is reduced by covering a Petri dish with a glass plate.

The mechanism that underlies the generation of the regular 2D hexagonal pattern is understood to be as follows: instead of being dissolved in water, the PFOB molecules contained in a droplet actually spread to form a fluid nanolayer floating on the water surface around the droplet. Simultaneously, this nanolayer evaporates slowly into air. As a result, the nanolayer spreads over a certain area that has a characteristic length scale. This scale is determined by the balance between the supply of PFOB molecules coming from the droplet and their evaporation from the nanolayer. Such evaporation maintains a flow in the nanolayer that is directed outwards. This type of repulsion, due to the intrinsic nature of dewetting transition, is also at work between droplets and the rim of the film, which drives the periodical ejection. Collision between the flows of the nanolayers issued from two neighbouring droplets will transform the kinetic energy per unit volume into pressure. This pressure would cause the observed repulsive force arising between droplets. Actually, when we covered the Petri dish with a glass plate just after having spread PFOB to reduce evaporation rate, the ordering of droplets became more irregular than that without the plate (Fig. 5a,c). This demonstrates that the driving forces for the formation of the dynamic pattern are not of thermodynamic origin (such as depletion force, for example), as they cannot operate in the absence of evaporation-generated motion, but are rather driven by nonlinear kinetics of the dewetting transition and evaporation under thermodynamically dissipative conditions. The rates of surface-spreading and evaporation of the fluorocarbon around a certain droplet can be evaluated as follows: the flow rate supplied by a droplet onto the surface is proportional to the length of a contact line between the PFOB layer and the water surface (∝2π*r*, where *r* is the radius of the droplet), while the rate of evaporation is proportional to the surface area of the nanolayer (∝*S*, where *S* is the area of the nanolayer). Considering a quasi-steady state where both rates are balanced, the area of the nanolayer is proportional to the radius of the droplet (*S*∝*r*). It is technically difficult to precisely evaluate the excluded area generated by a droplet. Nevertheless, we estimated this area based on a Voronoi diagram (Fig. 5a). The relationship between the radii of the droplets and the area of their individual Voronoi domains is shown in Fig. 5b. The red line in Fig. 5b demonstrates that the minimum size (marked by dark-blue circles) of the Voronoi domain linearly correlates with the droplet radius. This threshold value is thought to correspond to the excluded area of the nanolayer, while the threshold value when we covered the Petri dish is equivocal (Fig. 5d). This correlation supports the validity of our proposed mechanism based on evaporation-induced flow within the nanolayer (*S*∝*r*).

## Discussion

We describe a new synthetic system for the spontaneous creation and dynamic self-assembly of fluorocarbon droplets on water. This ensemble of droplets undergoes a transformation in a confined space between 1D arrays of microdroplets and 2D hexagonal arrays through periodical collective motion. This results in the formation of a hexagonal pattern that shows a unique collective cyclic shrinking and expanding behaviour, reminiscent of a flock of birds. These dynamics can be attributed to repulsive force between the droplets. An evaporation-induced surface flow, which is a quasi-2D flow of a PFOB nanolayer on the surface of water, may account for this phenomenon. This simple synthetic system that exhibits spatiotemporal behaviour appears to mimic collective behaviours found in nature and may serve as a model for the study of dynamically assembled systems. Furthermore, liquid fluorocarbons are generally immiscible with most organic solvents. Their characteristics are expected to provide new strategies for the design of systems of hybrid particles composed of mutually immiscible liquids that are expected to show novel non-equilibrium behaviour.

## Methods

### Chemicals

PFOB, C_8_F_17_Br, was obtained from Alliance Pharmaceutical Corp. (San Diego, CA) and thoroughly purified by repeated distillations and alumina column treatment.

### Observation of the dynamic behaviour of a PFOB droplet

A droplet of PFOB (8 μl) was deposited on the surface of deionized water (30 ml) in a Petri dish with a diameter of 91 mm (Fig. 2a). PFOB floats on water, even though PFOB is more dense (1.9 g ml^−1^) than water, because the high interfacial tension overcomes the growth of Rayleigh–Taylor instability. The behaviour of droplets was monitored at room temperature (20–25 °C) using an optical microscope (VX-6,000/5,000, Keyence Corp.) equipped with a universal zoom lens (VH-Z20UW, Keyence Corp.).

## Additional information

**How to cite this article:** Yamamoto, D. *et al*. The evolution of spatial ordering of oil drops fast spreading on a water surface. *Nat. Commun.* 6:7189 doi: 10.1038/ncomms8189 (2015).

## Supplementary Material

Supplementary Figures, Notes and References Supplementary Figure 1, Supplementary Note 1 and Supplementary References

Supplementary Movie 1 Dynamics of a PFOB droplet on a water surface. All of the movies have the same scale: 3078 μm×4104 μm.

Supplementary Movie 2 Evaporation of a cyclohexane film. All of the movies have the same scale: 3078 μm×4104 μm.

Supplementary Movie 3 Dynamics of a PFOB droplet at a low temperature. All of the movies have the same scale: 3078 μm×4104 μm.
