# Supplementary material for: The evolution of spatial ordering of oil drops fast spreading on a water surface
Source: Nat Commun. 2015 May 22;6:7189. doi: 10.1038/ncomms8189 (PMC4455131; doi:10.1038/ncomms8189)
Supplement: Supplementary Figures, Notes and References — Supplementary Figure 1, Supplementary Note 1 and Supplementary References [file ncomms8189-s1.pdf]

Supplementary Figure

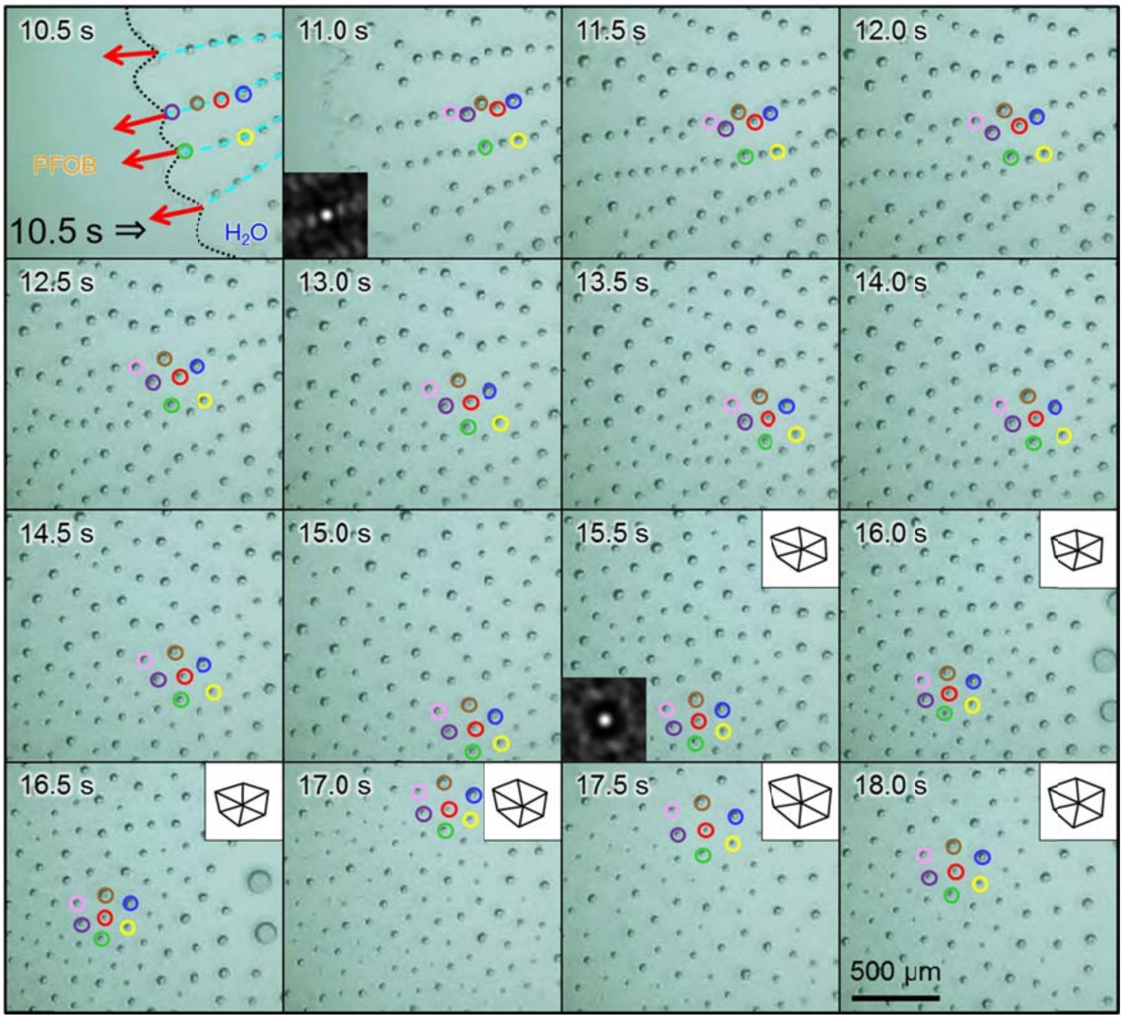

Supplementary Figure 1 Details of the spontaneous transformation from 1-D to 2-D ordering (time interval: 0.5 s) as shown in **Fig. 4c**. The ejected droplets show spatio-temporal collective behaviour. After cascade ejection (10.5–11.5 s), the aligned droplets rearrange into a 2-D pattern (11.5–15.5 s). The resultant 2-D pattern shows cyclic shrinkage and expansion of the whole population of droplets to eventually form a 2-D hexagonal pattern (15.5–18.0 s). Insets in snapshots at 15.5–18.0 s show hexagonal patterns formed by the seven neighbouring coloured droplets.

Supplementary Note 1

PFOB liquid initially spreads to form a thin film on a water surface. For a partial-wetting liquid film (liquid 1) on a liquid substrate (liquid 2), the critical initial thickness for dewetting is $h_{\mathrm{c}} = \sqrt{-S/(1/2\tilde{\rho}\, g)}$[1], where S, $\tilde{\rho} = \rho_1(1-\rho_1/\rho_2)$, ρ_1, and ρ_2 are the spreading coefficient, effective density, density of liquid 1 and density of liquid 2, respectively. When the thickness of a liquid film is less than the critical thickness, dewetting is caused by the generation of a small hole in the PFOB film (**Fig. 1**). At an early stage, the dewetting hole grows constantly and follows Culick's law $V = \sqrt{|S|\left(1-h^2/{h_{\mathrm{c}}}^2\right)/(2K\rho_1 h)}$, where V is velocity and $K = 2.0$[1]. During the dewetting of the contact line, the rim forms at the dewetting front. As the rim recedes, a linear array of droplets, or a pearling structure, (**Fig. 1b**), is formed through Rayleigh instability because of the competition between surface tension and the effect of gravity[2]. The connection between droplets on the rim and the film is unstable and will be broken as the rim recedes. The droplets left behind the dewetting rim are arranged along a line in the direction in which the contact line recedes. If we consider the evolution of the thin liquid film, the instability of the interface can be described using the following equation, which includes the effect of evaporation:

$$\frac{\partial h}{\partial t} \approx \nabla\{Q(h)\nabla[-\gamma\nabla^2 h - \Pi(h)]\} - \alpha h \qquad (1)$$

where h is the thickness of the film, $Q(h)$ is the mobility of the liquid, $\Pi(h)$ is the disjoining pressure in the film, γ is surface tension, and α is a constant for the evaporation rate. Under the assumption that $Q(h)$ is a constant and α is negligibly small, we can estimate the wavelength λ_{inst} with the maximum growth rate, or maximum instability, by taking the approximation of a linear stability analysis in one dimension, as follows:

$$\lambda_{\mathrm{inst}} \approx 2\pi\sqrt{\frac{2\gamma}{-\Pi'(h_0)}} \qquad (2)$$

Thus, we can expect pearling instability accompanied by the dewetting transition. Thus, in the initial stage of hole growth, pearling instability appears along the circumference of the hole. After the appearance of arranged humps, or alignment of the droplets, along the periphery of the hole, pearling instability propagated in the radial direction, as demonstrated in Fig. 1. As shown in our experiment, the actual phenomenon is somewhat more complicated and it is important to include the kinetic effect[3]. Further theoretical analysis is expected to reveal the mechanisms of the exotic phenomena reported in the present study.

Reference

1 Chen, Y. J., Nagamine, Y. & Yoshikawa, K. Self-propelled motion of a droplet induced by Marangoni-driven spreading. *Phys. Rev. E* 80, 016303 (2009).

2 de Gennes, P. G., Brochard-Wyart, F. & Quere, D. *Capillarity and wetting phenomena: Drops, bubbles, pearls, waves*. (Springer, 2004).

3 Thiele, U., Velarde, M. G. & Neuffer, K. Dewetting: film rupture by nucleation in the spinodal regime. *Phys. Rev. Lett.* 87, 016104 (2001).
